# Inclusion Complexes of β and HPβ-Cyclodextrin with α, β Amyrin and In Vitro Anti-Inflammatory Activity

**DOI:** 10.3390/biom9060241

**Published:** 2019-06-21

**Authors:** Walter Ferreira da Silva Júnior, Danielle Lima Bezerra de Menezes, Luana Carvalho de Oliveira, Letícia Scherer Koester, Patrícia Danielle Oliveira de Almeida, Emerson Silva Lima, Eduardo Pereira de Azevedo, Valdir Florêncio da Veiga Júnior, Ádley Antonini Neves de Lima

**Affiliations:** 1 Pharmacy Department, Universidade Federal do Rio Grande do Norte, Natal 59012-570, RN, Brazil; walterjuniornt@hotmail.com (W.F.d.S.J.); daniellelbmenezes@gmail.com (D.L.B.d.M.); farmaceuticalu@hotmail.com (L.C.d.O.); 2 Production and Drug Control Department, Universidade Federal do Rio Grande do Sul, Porto Alegre 90610-000, RS, Brazil; leticia.koester@ufrgs.br; 3 Laboratory of Biological Activity, Faculty of Pharmaceutical Sciences, Universidade Federal do Amazonas, Manaus 69077-000, AM, Brazil; patt_danielle@hotmail.com (P.D.O.d.A.); eslima75@gmail.com (E.S.L.); 4 Graduate Program of Biotechnology, Laureate International Universities (UnP), Natal 59056-000, RN, Brazil; azevedoep@hotmail.com; 5 Chemical Engineering Department, Military Institute of Engineering, Rio de Janeiro 22290-270, RJ, Brazil; valdir.veiga@gmail.com

**Keywords:** amyrin, cyclodextrin, inclusion complexes, anti-inflammatory

## Abstract

α, β amyrin (ABAM) is a natural mixture of pentacyclic triterpenes that has a wide range of biological activities. ABAM is isolated from the species of the Burseraceae family, in which the species *Protium* is commonly found in the Amazon region of Brazil. The aim of this work was to develop inclusion complexes (ICs) of ABAM and β-cyclodextrin (βCD) and hydroxypropyl-β-cyclodextrin (HPβCD) by physical mixing (PM) and kneading (KN) methods. Interactions between ABAM and the CD’s as well as the formation of ICs were confirmed by physicochemical characterization in the solid state by Fourier transform infrared (FTIR), scanning electron microscopy (SEM), X-ray diffraction (XRD), thermogravimetry (TG) and differential scanning calorimetry (DSC). Physicochemical characterization indicated the formation of ICs with both βCD and HPβCD. Such ICs were able to induce changes in the physicochemical properties of ABAM. In addition, the formation of ICs with cyclodextrins showed to be an effective and promising alternative to enhance the anti-inflammatory activity and safety of ABAM.

## 1. Introduction

The species *Protium heptaphyllum* (Burseraceae) is popularly known in Amazon, Brazil as “breu-branco” and “almecegueira” [1]. From the trunk of its trees is obtained a natural exuded oil-resin that is rich in volatile monoterpenes and triterpenes, especially α and β amirin (ABAM) (C_30_H_50_O) [2,3]. Having a wide range of biological activities, triterpenes have aroused clinical interest [4]. ABAM is a well-known natural mixture of isomeric triterpenes whose pharmacological properties include anti-inflammatory [5], gastroprotective [6], antitumor [7], anxiolytic [8] and hepatoprotective [6]. The anti-inflammatory activity of ABAM has been demonstrated in different experimental models through the inhibition of the release of pro-inflammatory cytokines (IL-6, TNF-α and IL-1β) and the enzyme myelopereroxidase [6,9].

Considering that ABAM has a pronounced lipophilicity and that in vivo studies using rats have shown low bioavailability for both its isolated form (0.86%) and its extract (3.83%) [10], it becomes important to develop a system that is capable of enhancing the physicochemical properties of ABAM while making this new chemical entity feasible to be used as a new drug.

Cyclodextrins (CDs) are cyclic oligosaccharides of a variable number of glucose units, linked by α-(1,4) glycosidic bonds. They are characterized by a hydrophobic cavity and a hydrophilic exterior, and for this reason, CDs are able to host hydrophobic molecules in their interior, therefore, forming the so-called inclusion complexes (ICs) [11]. Some drugs are already marketed in Europe, Japan, United States and Brazil as complexes with cyclodextrins, which includes dexamethasone, nimesulide, omeprazole, piroxicam, indomethacin and itraconazole [12,13].

CDs have the ability to bind to the host molecule by non-covalent interactions in both aqueous and solid states, where such interactions have been capable of modifying the physicochemical properties of the bound molecule, which results in greater safety, solubility, stability and bioavailability [14]. Several studies have demonstrated the ability of CDs in enhancing the safety, bioavailability and pharmacological activity of substances with anti-inflammatory properties [15,16,17,18].

The aim of this work was to develop inclusion complexes (ICs) of ABAM with β-cyclodextrin (βCD) and hydroxypropyl-β-cyclodextrin (HPβCD) with the purpose of improving the physicochemical properties of ABAM. In addition, the in vitro anti-inflammatory activity of ABAM-CDs complexes was investigated using lipopolysaccharide-stimulated mouse macrophage cell line J774.

## 2. Materials and Methods

### 2.1. Materials

ABAM was obtained following the protocol described by Silva-Júnior et al. [19], from commercially available Burseraceae oi-resin from Manaus, Central Amazon (Brazil), where *Protium heptaphyllum* is the endemic species. The sample of ABAM used at this study is the same one that was previously purified, isolated and characterized by gas chromatography coupled with mass spectrometry (GCMS) and ^1^H and ^13^C nuclear magnetic ressonance (NMR), which presents more than 99% of purity and a correlation between the isomers α and β amyrin (as determined by NMR and confirmed by GCMS) of 2.64:1. βCD and HPβCD were purchased from Sigma-Aldrich^®^ (St. Louis, MO, USA). Purified water (conductivity less than 1.3 μS/cm) obtained by reverse osmosis was used in all experiments. Analytical grade reagents were also used.

### 2.2. Preparation of ICs

ICs were prepared by physical mixing (PM) and kneading (KND). Both methods were performed following a 1:1 molar ratio between ABAM and the CD, considering the molecular weight of ABAM as 426.72 g mol^−1^ [20,21] and those of βCD and HPβCD as 1134.98 and 1375.37 g mol^−1^, respectively.

#### 2.2.1. Physical Mixture (PM)

ABAM and either βCD or HPβCD were weighed following the 1:1 molar ratio and mixed with mortar and pestle. The resulting powder mixture was kept in desiccator until further analysis.

#### 2.2.2. Kneading (KN)

The kneading method was performed following a previously reported procedure [22] with some minor modifications. ABAM and either βCD or HPβCD were weighed separately followed by mixing with mortar and pestle. Purified water and acetone were then added to the mixture (approximately 10 mL of purified water for each 1 g of the ABAM/CD mixture) followed by mixing using mortar and pestle. The obtained samples were dried in an oven at 60 °C until constant weight. The resulting powder was kept in desiccator until further analysis.

### 2.3. Physicochemical Characterization

#### 2.3.1. Fourier Transform Infrared (FT-IR)

Fourier-transform infrared (FT-IR) (4000–400 cm^−1^) were obtained as KBr pellets on a Prestige-21 FT-IR spectrophotometer (Shimadzu, Kyoto, Japan). The number of scans was 16 and the resolution was 4 cm^−1^.

#### 2.3.2. Scanning Electronic Microscopy (SEM)

Samples were fixed in stubs using double-sided carbon tape and analyzed on a Hitachi Scanning Electron Microscope (Tokyo, Japan), with a minimum magnification of 400× and maximum of 2000×. The photomicrographs were obtained at 15 kV under reduced pressure.

#### 2.3.3. Powder X-Ray Diffraction (XRD)

The XRD profiles of the samples were obtained on a Bruker D2 Phaser diffractometer (Billerica, MA, EUA), using CuKα radiation (λ = 1.54 Å) with Ni filter. The analysis was performed with a step of 0.02°, 10 mA current, 30 kV voltage and a Lynxeye detector.

#### 2.3.4. Thermogravimetry (TG)

Thermogravimetric analyses were carried out using a Shimadzu^®^ TGA-50 (Kyoto, Japan). About 2 mg of each sample was weighted, sealed in aluminum crucibles and heated at a rate of 10 °C·min^−1^ within the temperature range of 30–600 °C under a dynamic atmosphere of N_2_ (50 mL·min^−1^). A blank aluminum crucible was used as reference during the analysis. Prior to the test, the instrument was calibrated using a standard CaC_2_O_4_·H_2_O.

#### 2.3.5. Differential Scanning Calorimetry (DSC)

Differential scanning calorimetry (DSC) analyses were carried out using a Shimadzu^®^ DSC 50 apparatus (Kyoto, Japan)), where 2 mg of each sample was hermetically sealed in aluminum crucibles with a heating rate of 10 °C·min^−1^ within the temperature range of 30–500 °C under a dynamic atmosphere of N_2_ (50 mL·min^−1^). A blank aluminum crucible was used as reference during the analysis. Prior to the test, enthalpic calibration was performed using indium (melting point: 156.6 °C; ΔHm = 28.54 J·g^−1^) and zinc (melting point: 419.6 °C) as standards.

### 2.4. In Vitro Anti-Inflammatory Study

The anti-inflammatory activity of ABAM alone and as ICs with cyclodextrins was evaluated using an in vitro inflammation model based on the quantification of nitric oxide (NO^−^) produced by lipopolysaccharide-stimulated mouse macrophage cell line J774 (LPS), in which the levels of nitrite was quantified in culture medium by the Griess reaction. Cells were cultured in 24-well culture plates at a density of 1 × 10^6^ cells / mL and incubated in an oven at 37 °C at a 5% CO_2_ atmosphere for 2 h for adhesion of the cells to the plate. Further, the cells were stimulated with 1 mL of LPS for 60 min. After stimulation, the cells were treated with ABAM alone and as ICs with either βCD or HPβCD at a concentration of 20 μg/mL. The plates were again incubated under the same conditions for 24 h. Finally, the supernatant was collected for quantification of NO.

#### Quantification of Nitric Oxide (NO^−^)

NO^−^ production was determined by spectroscopic quantification of nitrite levels by Griess reaction [23] in the supernatant of the LPS-stimulated J774 macrophages cells treated with ABAM alone and as ICs with either βCD or HPβCD.

In a 96-well microplate, aliquots of 50 μL of the supernatant were collected and added to 100 μL of Griess reagent (1:1 mixture of 1% sulfanylamine dihydrochloride and 0.1% N-[1-naphthyl] ethylenediamine), followed by incubation for 10 min at room temperature and protected from light. The absorbance was determined spectroscopically at 570 nm on a microplate reader.

### 2.5. Cell Viability Assay

Cell viability was assessed through MTT assay. J774 macrophage cells were plated into 96-well microplates with a cell density of approximately 3.4 × 10^−3^ cells/well. ABAM alone or as ICs were added to each well at a concentration of 20 μg/mL followed by plate incubation for 24 h in oven at 37 °C and atmosphere of 5% CO_2_. Centrifugation was performed followed by removal of the supernatant and immediately replenished with fresh medium containing MTT reagent (Sigma, St. Louis, MO, USA, 0.5 mg/mL). After 4 h of incubation under the same conditions, the supernatant was removed and 150 μL of DMSO was added for the dissolution of MTT-formazan followed by quantification on a microplate reader (DTX 880 Multimode Detector, Beckman Coulter Inc., Fullerton, CA, USA) at 540 nm. Cell viability assay was performed in triplicate.

### 2.6. Statistical Analysis

All results were presented as mean ± standard deviation. Two-way ANOVA was performed with Bonferroni post-test using GraphPad Prism version 5.0 (GraphPad Software, San Diego, CA, USA). Values of *p* less than 0.05 were considered as statistically significant.

## 3. Results

### 3.1. Physicochemical Characterization

#### 3.1.1. Fourier Transform Infrared (FT-IR)

**Figure 1:**
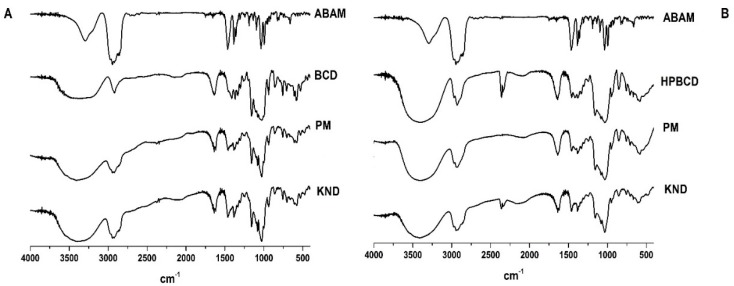
Fourier transform infrared (FT-IR) spectra of α, β amyrin (ABAM), β-cyclodextrin (βCD), hydroxypropyl-β-cyclodextrin (HPβCD), and the corresponding inclusion complexes (ICs) obtained with βCD (**A**) and HPβCD (**B**) by physical mixture (PM) and kneading (KND) methods.

The FT-IR spectra of each individual sample (ABAM, βCD and HPβCD) and ICs are shown in Figure 1. Based on previous reports [24], the ABAM spectrum presents a high intensity band at 2800 cm^−1^ that has been associated with the axial deformation of the C-H bonds in cyclic chains. The band at the 3250–3400 cm^−1^ region is attributed to the hydroxyl group (O-H) attached to a cycloalkane chain and that of the 1031–1100 cm^−1^ region is associated with C-O bond vibration. Finally, another characteristic band is found in the 1400–1350 cm^−1^ region, which is due to the angular deformation of the dimethyl and methyl duplet [19].

The spectrum of βCD has characteristic bands at 3300 cm^−1^ (stretching vibrations of OH bond), 2925 cm^−1^ (CH stretch vibrations), 1151 cm^−1^ and 1023 cm^−1^ (symmetrical and asymmetrical stretching vibrations of C-O-C, respectively), as previously reported [25,26]. The HPβCD spectrum shows characteristic bands in the regions of 3355 cm^−1^ (OH stretching vibrations), 2922 cm^−1^ (CH bond stretching vibrations), 1151 cm^−1^ and 1080 cm^−1^ (stretching vibrations of C-H and C-O, respectively), as reported by Medarević’s [27].

According to the FT-IR spectra shown in Figure 1A,B, the characteristic bands of βCD and HPβCD are predominant (axial deformation of the OH bond) around 3355 to 3300 cm^−1^. The spectra of ABAM-βCD obtained by PM and KND (Figure 1A) show similarities to the spectrum of βCD, which seems to indicate that ABAM spectrum has been hindered in both spectra of PM and KND [28]. However, the bands around the 3300 cm^−1^ region in the KND spectrum showed higher intensity than those of ABAM alone and PM. It seems that the KND method facilitated the inclusion of ABAM in the βCD cavity in which hydrogen bonds might have been formed between these two.

Based on the spectra of ABAM-HPβCD obtained by PM and KND (Figure 1B), the bands attributed to ABAM were almost completely obscured by HPβCD bands, which are very intense and broad. In both spectra, the major ABAM bands (3400–3250 cm^−1^, 2800 cm^−1^, 1100–1041 cm^−1^, 1450–1350 cm^−1^) disappeared and since no new bands have been identified in both spectra, it seems to rule out the possibility of formation of new chemical bonds [29].

Therefore, the results show that both βCD and HPβCD interact with ABAM, regardless of the preparation method (PM and KND), which indicates that even in solid state both CD’s have the ability to form complexes with ABAM.

#### 3.1.2. Scanning Electronic Microscopy (SEM)

**Figure 2:**
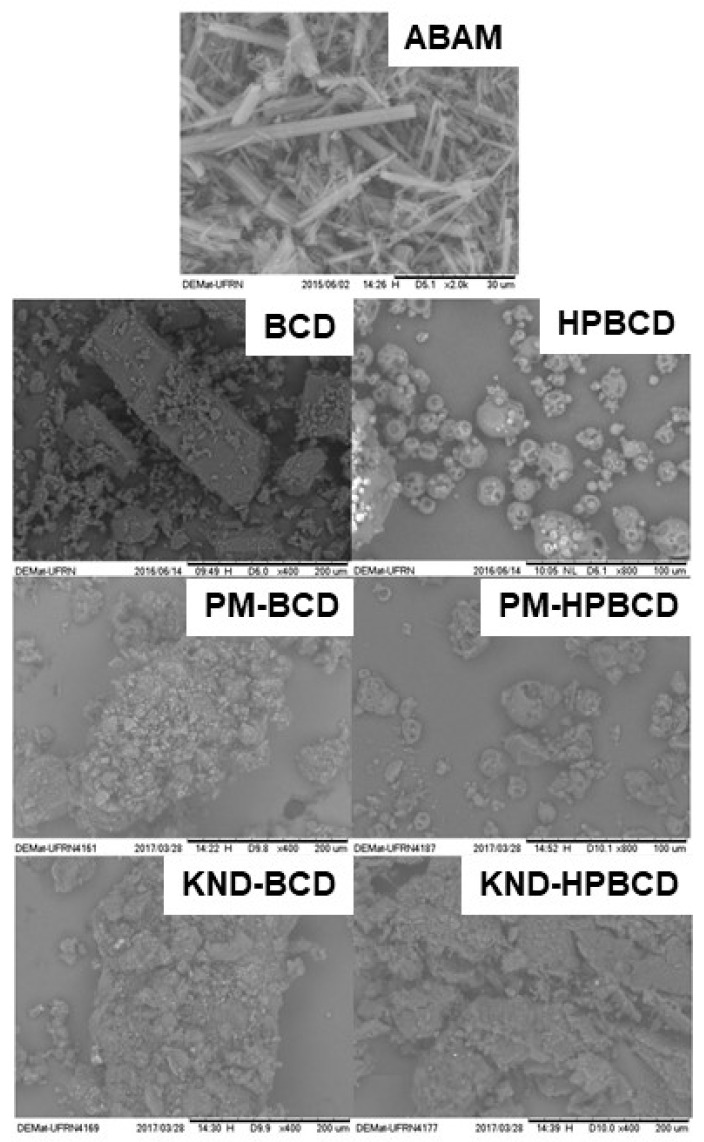
Scanning electron microscopy (SEM) micrographs of ABAM, βCD, HPβCD as well as ABAM-βCD and ABAM-HPβCD prepared by PM and KND methods with magnifications of 400× and 2000×.

The surface morphology of each individual compound and their respective ICs are presented in Figure 2. The ABAM’s surface is characterized by well-defined acicular structures with regular shapes and a three-dimensional appearance, which are characteristic of a crystalline compound [19]. βCD presents particles with crystalline structure in parallelogram format, as observed by Bulani [30]. On the other hand, HPβCD presents as amorphous and spherical particles, as observed by Melo [31].

The photomicrographs of ABAM-βCD obtained by PM and KND show aggregates of different sizes with porous surfaces, whose appearance differs from that of the βCD particles. These morphological changes might be an evidence of drug-CD interaction, as reported elsewhere [32].

On the other hand, the surface morphology of ABAM-HPβCD prepared by PM was not altered in comparison with HPβCD and ABAM. Therefore, such macroscopic observations seem to evidence the presence of each of the constituents with maintenance of their original morphology, with ABAM crystals adhered to the surface of HPβCD, which seems to indicate that no interactions took place between ABAM and HPβCD. However, the photomicrograph of ABAM-HPβCD prepared by KND shows changes in the morphological aspect of the surface, with loss of the original spherical shape typical of HPβCD as well as the characteristic crystalline structure of ABAM.

#### 3.1.3. Powder X-ray Diffraction (XRD)

**Figure 3:**
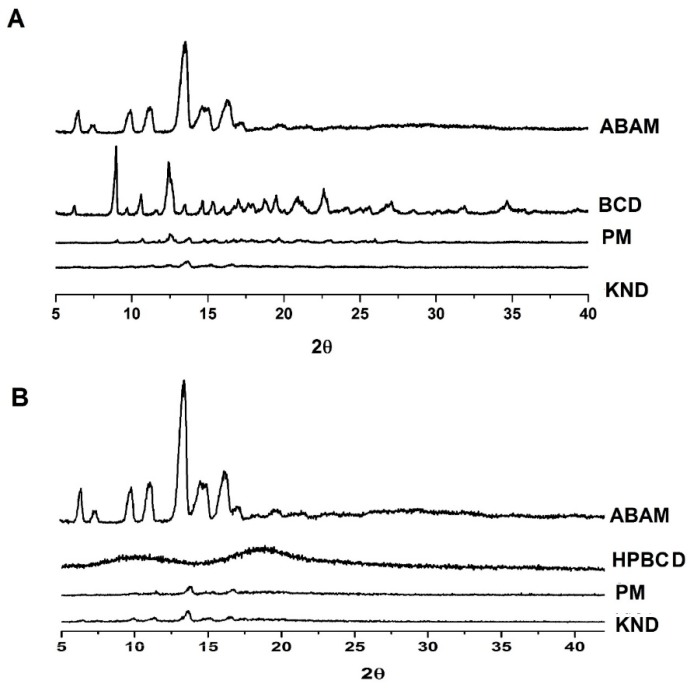
X-ray diffraction patterns of ABAM and its ICs. (**A**) βCD alone and as ABAM-βCD obtained by PM and KND, (**B**) HPβCD alone and as ABAM-HPβCD obtained by PM and KND.

The diffraction profiles of ABAM, βCD and HPβCD and their ICs are shown in Figure 3. A crystalline powder consists of a solid whose three-dimensional structure is capable of refracting X-rays with very characteristic and well-defined reflection peaks [33]. The diffraction profile of ABAM shows intense crystalline reflection at 13°. In addition, other secondary reflections are observed at 6°, 10°, 11°, 14° and 16°, which are in accordance with Silva-Júnior [24] and characterizes ABAM as a crystalline compound. The diffraction profile of βCD presents crystal reflections at 8°, 10°, 12°, 19°, 20° and 22° in addition to secondary crystalline reflections. On the other hand, HPβCD presents two wide diffraction halos, which are characteristic of amorphous materials.

The diffractograms of ABAM-βCD obtained by PM and KND methods (Figure 3A) show a significant reduction in the intensity of the crystalline reflections attributed to each individual component. It is worth to point out that the diffractogram of ABAM-βCD obtained by KND presents a greater suppression of the crystalline reflections, which seems to indicate the formation of a new crystalline phase and, therefore, the formation of a three-dimensional arrangement different from those of ABAM and βCD alone [34].

Similarly, ABAM-HPβCD prepared by PM and KND (Figure 3B) showed distinct diffraction profiles in comparison to those of ABAM and HPβCD. However, a suppression of the crystalline reflections of ABAM was observed, which seems to indicate an amorphization of the newly formed complex. Nevertheless, discrete diffraction peaks are still observed in the ABAM-HPβCD, which may result from the presence of the non-complexed ABAM.

Therefore, it seems likely to infer that interaction took place between ABAM and both βCD and HPβCD as the systems obtained with these CDs presented different diffraction profiles in comparison to that of each individual compound.

#### 3.1.4. Thermogravimetry (TG)

**Figure 4:**
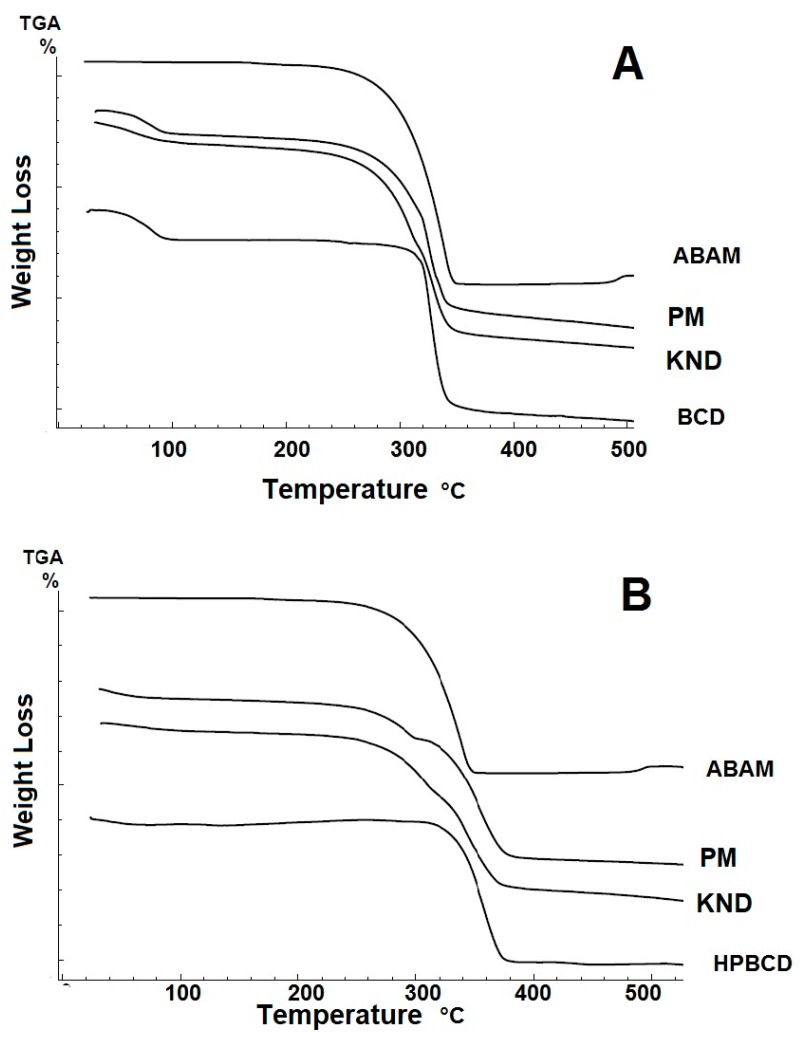
Thermogravimetry (TG) curves for ABAM, βCD, HPβCD and their respective ICs. (**A**) TG curves for ABAM-βCD obtained by PM and KND. (**B**) TG curves for ABAM-HPβCD obtained by PM and KND.

The TG curves and mass losses for ABAM, βCD, HPβCD and their respective ICs are shown in Figure 4 and Table 1. The thermogravimetric curve for ABAM shows only one well-defined stage of mass loss which is evidenced by derivative TG (DTG), which seems to be related to its volatilization. The mass loss percentage (Δm%) was approximately 99.5%, where the initial and final mass loss temperatures were 232 and 347 °C, respectively.

The TG curve for βCD (Figure 4A) presents two stages of mass loss, evidenced by the DTG curve, where the first one showed 13.60% of mass loss due to the release of water molecules from its cavity, as observed by Menezes [35]. The second stage begins after 300 °C and is due to its degradation. The curve for HPβCD (Figure 4B) shows a mass loss of 3.9% in the temperature range of 30–150 °C, which might be due to the release of water molecules. After 300 °C, HPβCD undergoes degradation.

The TG curves for ABAM-βCD obtained by PM and KND present initial mass loss between 25 °C and 120 °C, which is related to water loss from the βCD cavity. The percentage of mass loss (Δm) for PM and KND was 10.45% and 9.65%, respectively, within this temperature range. Therefore, a decrease in the initial loss of water was observed for both systems in comparison to that of βCD. At higher temperatures, no evidence of complexation could be found because the degradation temperature of ABAM was very close to that of βCD.

Regarding the loss of mass of ABAM-HPβCD in the initial temperature range, 5.5% and 3.81% of mass losses were observed for PM and KND, respectively. As previously observed, ABAM-HPβCD obtained by PM loses more mass than HPβCD in the temperature range of 25–120 °C, which seems to indicate no interaction between ABAM and HPβCD. On the other hand, the ICs obtained by KND showed lower mass loss when compared to HPβCD and ABAM-HPβCD prepared by PM.

#### 3.1.5. Differential Scanning Calorimetry (DSC)

**Figure 5:**
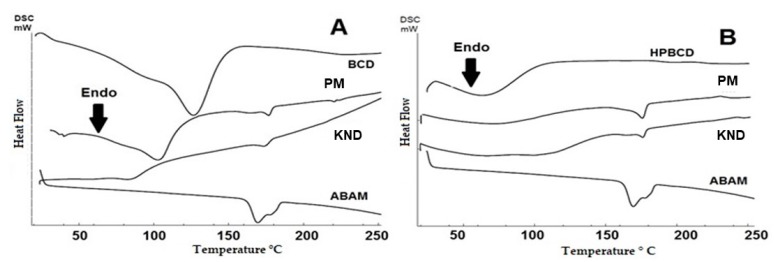
Differential scanning calorimetry (DSC) curves for ABAM, CDs and their respective ICs obtained with βCD (**A**) and HPβCD (**B**) by PM and KND.

The DSC curves for the individual compounds and the obtained ICs are shown in Figure 5. The DSC curve for ABAM shows an endothermic event between 158 and 185 °C with a peak at 170 °C and enthalpy (ΔH) of 46 J·g^−1^ which corresponds to the melting of the triterpenic mixture. An endothermic event in the range of 34–155 °C is observed for βCD (Figure 5A), which corroborates with the results of the thermogravimetric analysis regarding the loss of water molecules. This same phenomenon is also observed in the DSC curve for HPβCD (Figure 5B), whose endothermic event appears in the temperature range of 34–110 °C.

The curves for ABAM-βCD (Figure 5A) demonstrated that both PM and KND methods led to a decrease of the melting event, as can be seen with the decay of the enthalpy, whose values for ABAM-βCD prepared by PM and KND are 4.54 J/g and 3.63 J/g, respectively, suggesting a change in the crystalline state as a result of the complexation.

On the other hand, the DSC curve for ABAM-HPβCD (Figure 5B) shows that both PM and KND decreased the endothermic event related to the melting of ABAM, whose enthalpy values were 9.61 J/g and 5.70 J/g. This finding seems to indicate that some interaction between ABAM and HPβCD took place. In addition, a decrease in the water loss event of HPβCD is observed, which is another indication that complexation took place after the displacement of water molecules from the internal cavity of HPβCD.

### 3.2. In Vitro Anti-inflammatory Study

**Figure 6:**
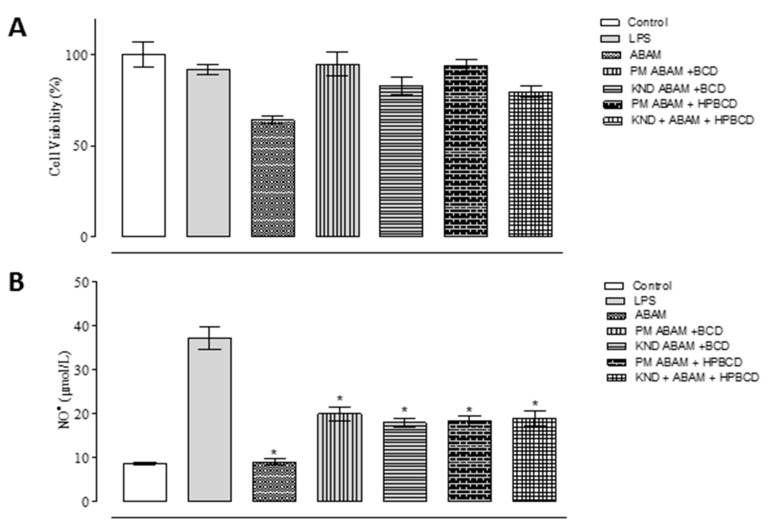
In vitro anti-inflammatory activity in lipopolysaccharide-stimulated mouse macrophage cell line (LPS)-stimulated J774 macrophages for quantification of NO- and cell viability by MTT in cells treated with ABAM and its inclusion complexes with βCD and HPβCD at a concentration of 20 μg/mL after 24 h. (**A**): Percentage of cell viability after 24 h of treatment with ABAM and its ICs; (**B**): Inhibitory percentage of each individual compounds and the respective ICs towards the production of nitric oxide.

In vitro models of inflammation based on LPS stimulation have been used for searching new anti-inflammatory drugs [36]. The in vitro anti-inflammatory activities of ABAM as well as ABAM-βCD and ABAM-HPβCD are shown in Figure 6A. When comparing the inhibitory activity towards nitric oxide production of ABAM, ABAM-βCD and ABAM-HPβCD in relation to the control groups (positive control: Stimulated cells with LPS, negative control: Cells without LPS stimulation), it seems that ABAM, alone and the ICs, present anti-inflammatory activity.

The inhibitory capacity of ABAM-βCD obtained by KND was about 60.5%, while ABAM-HPβCD prepared by PM was around 59.8%. These results imply that ABAM complexation with both cyclodextrins was able to enhance its safety and anti-inflammatory activity, as ABAM alone and complexed with CDs were tested at the same concentration of 20 μg/mL.

### 3.3. Cell Viability Assay

The cell viability of the J774 macrophages was analyzed by MTT assay as shown in Figure 6B. The results show that more than 70% of cell viability was observed with the group treated with ABAM alone. On the other hand, ABAM-βCD and ABAM-HPβCD increased the cellular viability by 80–100%, implying that the formation of ICs was able to decrease the activity of ABAM against the proliferation of J774 macrophages. It is further observed that even with LPS promoting cell viability of approximately 60%, the cells treated with ABAM (alone and complexed with CDs) still showed significant viability. Therefore, it is suggested that ABAM and its inclusion complexes are classified as non-toxic.

## 4. Discussion

Complexation with cyclodextrins is one of the most commonly used strategy to increase the solubility, stability and safety of poorly soluble molecules [11]. Thus, the physicochemical characterization of such complexes is of utmost importance in order to prove that complexation has taken place. In this current study, physicochemical characterization in addition to in vitro assays were carried out with the purpose of evaluating the feasibility of the formation of inclusion complexes between ABAM and cyclodextrins as well as the resulting improvement of its anti-inflammatory activity.

FTIR spectroscopy allows the identification of vibrational patterns of CDs and host molecules as well as changes in the characteristic bands of the ligand’s vibrational pattern such as disappearance, widening, changes in peak intensity or deviations in their wavenumbers. These changes may be a strong indicative of interaction between the host molecule and the CDs [37]. When an inclusion complex (IC) is formed between a molecule and CD, this can mask or hinder the characteristic peaks of the host molecule, especially when the complexation occurs within the internal cavity of the CD [38]. In this study, the characteristic bands of ABAM were masked as a result of ABAM-CDs complexation through PM and KND methods. The masking of the bands attributed to the host molecule, even when the drug-CD complex is prepared by simple PM, usually means that the drug is complexed inside the CD cavity [39]. A similar result was reported by Quintans [40], where a sapogenin was complexed with βCD and the bands attributed to the sapogenin were masked after the IC was obtained by PM.

SEM is widely used to morphologically identify the formation of inclusion complexes due to the fact that such complexation usually involves changes in the surface, particle size and appearance of the parent drug [41]. When a drug is complexed within the CD cavity, the morphology of the obtained IC tends to be distinct from that of each individual component [42,43]. As shown in Figure 2, the morphological aspects of both ABAM- βCD and ABAM- HPβCD were markedly different from those of ABAM, βCD and HPβCD.

XRD determines the crystalline nature of solids and therefore, has been used for the characterization of ICs [44]. By comparing the diffractograms of each individual substance with those of the corresponding ICs one can infer that complexation has taken place if changes between the diffractograms are observed [45,46]. In this current work, the characteristic crystalline profile of ABAM was changed after complexation with CDs by both methods of preparation, whose decrease in the crystalline profile might result in an enhancement of the ABAM’s aqueous solubility.

Thermal analyses aim to investigate the thermal behavior of compounds submitted to variations of heating and cooling in a controlled manner as defined in 2006 by the International Confederation for Thermal Analysis and Calorimetry [47]. TG evaluates mass losses due to heating as a function of time and therefore, has been used to identify the formation of ICs as evident changes in the mass loss profile are observed when a CD complexes with a host molecule [37]. On the other hand, DSC is used to identify melting, degradation and recrystallization processes. In most cases, molecules in the crystalline state, such as ABAM, exhibit a melting event that can be evidenced as an endothermic peak. When ICs are formed, this endothermic peak disappears due to the loss of the crystalline structure as a result of the complexation [37,48]. Even though the ICs still present a small event related to the ABAM’s melting, the observed event is of lower intensity, as confirmed by the low enthalpy, therefore, it can be suggested that such complexation favored a greater thermal protection to ABAM.

The results imply that the formation of inclusion complexes between ABAM and the two cyclodextrins improved the physicochemical properties of ABAM. Therefore, the in vitro anti-inflammatory model and the MTT assay were performed with the purpose of evaluating the viability of these inclusion complexes.

Inflammation is a natural and pathophysiological response of the body against lesions and infectious agents [49]. However, inflammation becomes harmful when the process progresses and becomes a chronic condition, which usually requires pharmacological intervention. Nitric oxide (NO^●^) is one of the main mediators of inflammation and its overproduction is associated with chronic inflammatory diseases. Therefore, NO^●^ has been used as the main marker of inflammation.

The MTT assay is commonly used to assess cell viability and proliferation by quantifying a blue-violet product called formazan crystal that is formed by enzymatic reduction in the cytoplasm of viable cells, which means that the higher its quantification, the greater the cell viability [50]. In this study, it was demonstrated that ABAM at the concentration of 20 μg/mL was able to inhibit the production of nitric oxide in LPS-stimulated macrophages J774 cells. However, considering that the inclusion complexes were obtained at a molar ratio of 1: 1 (ABAM:CD), we can infer that ABAM-CD has a higher NO^●^ inhibition activity in comparison with ABAM alone due to the fact that its concentration is twice of that in the ABAM-CD system, which means that although the concentration of ABAM in each sample was 20 µg·mL^−1^, and its concentration in the ICs was around 10 µg·mL^−1^. In addition, the MTT assay showed that all the analyzed samples did not interfere significantly in the cell viability, which seems to indicate lack or very low toxicity.

Thus, the formation of inclusion complexes with cyclodextrins seems to be an effective and promising alternative to enhance the safety of ABAM.

## 5. Conclusions

This study demonstrated that inclusion complexes between ABAM and βCD and HPβCD were successfully obtained by PM and KND methods, which were evidenced by the physicochemical characterization. In addition, it was observed that both ABAM and its inclusion complexes can be considered as non-toxic and that such complexation increased the anti-inflammatory activity of ABAM. It is of utmost importance to conduct further studies in order to identify possible incompatibilities with excipients used in the manufacture of pharmaceutical dosage forms.

## Figures and Tables

**Table 1:** Loss of mass of ABAM, βCD, HPβCD and their respective ICs within the temperature range of 25–120 °C.

Samples	∆m_1_ (%)
	25–200 °C
**ABAM**	0.02
**βCD**	13.60
**PM (βCD)**	10.45
**KN (βCD)**	9.65
**HPβCD**	3.93
**PM (HPβCD)**	5.5
**KN (HPβCD)**	3.81
